# Characterization of heterozygosity-rich regions in Italian and worldwide goat breeds

**DOI:** 10.1038/s41598-023-49125-x

**Published:** 2024-01-02

**Authors:** Giorgio Chessari, Andrea Criscione, Donata Marletta, Paola Crepaldi, Baldassare Portolano, Arianna Manunza, Alberto Cesarani, Filippo Biscarini, Salvatore Mastrangelo

**Affiliations:** 1 Dipartimento Agricoltura, Alimentazione e Ambiente, University of Catania, Via Santa Sofia 100, 95123 Catania, Italy; 2 Dipartimento Scienze Agrarie e Ambientali, Produzione, Territorio, Agroenergia, University of Milan, Via Giovanni Celoria 2, 20133 Milan, Italy; 3 Dipartimento Scienze Agrarie, Alimentari e Forestali, University of Palermo, Viale delle Scienze, 90128 Palermo, Italy; 4 CNR, Institute of Agricultural Biology and Biotechnology (IBBA), Via Bassini 15, 20133 Milan, Italy; 5 Dipartimento di Agraria, University of Sassari, Viale Italia 39, 07100 Sassari, Italy; 6 Animal and Dairy Science Department, University of Georgia, 425 River Road, 30602 Athens, GA USA

**Keywords:** Biodiversity, Animal breeding

## Abstract

Heterozygosity-rich regions (HRR) are genomic regions of high heterozygosity, which may harbor loci related to key functional traits such as immune response, survival rate, fertility, and other fitness traits. This study considered 30 Italian and 19 worldwide goat breeds genotyped with the Illumina GoatSNP50k BeadChip. The aim of the work was to study inter-breed relationships and HRR patterns using Sliding Window (SW) and Consecutive Runs (CR) detection methods. Genetic relationships highlighted a clear separation between non-European and European breeds, as well as the north–south geographic cline within the latter. The Pearson correlation coefficients between the descriptive HRR parameters obtained with the SW and CR methods were higher than 0.9. A total of 166 HRR islands were detected. CHI1, CHI11, CHI12 and CHI18 were the chromosomes harboring the highest number of HRR islands. The genes annotated in the islands were linked to various factors such as productive, reproductive, immune, and environmental adaptation mechanisms. Notably, the Montecristo feral goat showed the highest number of HRR islands despite the high level of inbreeding, underlining potential balancing selection events characterizing its evolutionary history. Identifying a species-specific HRR pattern could provide a clearer view of the mechanisms regulating the genome modelling following anthropogenic selection combined with environmental interaction.

## Introduction

In diploid organisms, single nucleotide differences observed between paternal and maternal chromosomes are called heterozygous sites^1^. With the continued development and cost reduction of high-throughput DNA sequencing and genotyping technologies, researchers have powerful tools for studying animal genomes through whole-genome molecular markers. However, little has been done to analyze genomic aspects of heterozygosity in livestock populations^2^, and the limited evidence suggests that increased heterozygosity over time may be attributed to selection^3^. Heterozygosity-rich regions (HRR), also known as runs of heterozygosity (ROHet), are a recently emerged analytical concept and refer to regions of consecutive heterozygous sites detected between paternal and maternal chromosomes in diploid organisms^4^. The analysis of HRR aims to identify genomic regions with high genetic variability, to provide information about the populations’ genetic diversity and evolutionary history^5^, as well as to identify specific segments in the genome where increased genetic diversity could be beneficial^6,7^. HRR may harbor loci associated to key functional traits such as immune response, survival rate, fertility and other fitness traits^8^ and avoid the deleterious effects of harmful homozygous genotypes^9–11^. Williams et al.^4^ first introduced the concept of HRR in livestock and suggested that some of these regions were under balancing selection and contained recessive lethal mutations in cattle. Along this line, further studies were subsequently carried out in cattle^6,7^, horses^3^, sheep^8,12^ and pigs^9,13,14^, but less commonly in goat species^15^. Goats (*Capra hircus*) were domesticated around 10,500 years ago in the Fertile Crescent^16^. After dispersion from its center of domestication, this species has undergone an intense adaptation process and occupied different agroecological areas worldwide^17^. Different breeds and goat strains have been selected for milk, meat and fiber production, playing an important role in the livestock sector around the world. Given the wide range of genetic variability and its ability to populate very different geographical areas and climates and produce in conditions of low anthropogenic input, the goat species is the best for studying genetic diversity and adaptation^18,19^. Thanks to these features, this species provides one of the most suitable models for understanding the patterns and distribution of HRR. Furthermore, Italy is the European country with the highest number of goat breeds, and it provides a precious reservoir of genetic diversity shaped by its varied history, environment, climate, and farming traditions^20^. Therefore, in this study, we investigated the genomes of Italian and worldwide goat breeds, to detect and characterize their HRR patterns and reveal regions of heterozygosity (HRR islands) which contain candidate genes related to specific traits.

## Results

### Genetic diversity indices

The genetic diversity indices were calculated per breed and then averaged per geographical group (see Table 1 for details on the dataset). The summary statistics showed a high variability all over the breeds (see Supplementary Table S1). On average, Asian breeds revealed the lowest values of expected (*H*_E_ = 0.340) and observed (*H*_O_ = 0.332) heterozygosity, and the highest average level of inbreeding (0.243). Conversely, the Turkish breeds (KIL and KLS) deviated from the Asian trend and showed values in contrast (*H*_O_ and *H*_E_ higher than 0.396 and inbreeding lower than 0.091). Similarly, African breeds showed a mean *H*_O_ of 0.371 and a mean *H*_E_ of 0.366, but a notable lower *F*_IS_ equal to 0.152. All European breed groups (Europe, Alpine arch, and Italy) reported average heterozygosity indices higher than 0.386 and average inbreeding coefficient lower than 0.121. Values of the Italy group of breeds spanned from ARG (*H*_O_ = 0.416, *H*_E_ = 0.412 and *F*_IS_ = 0.051) to MNT_I (*H*_O_ = 0.271, *H*_E_ = 0.263 and *F*_IS_ = 0.381) that also represented the range of the whole dataset. ARG, MLG, and JON were the breeds with the highest *H*_O_ (0.416, 0.415, and 0.415, respectively), while RCC, ARG, and M×S showed the highest *H*_E_ (0.414, 0.412, and 0.412, respectively). The lowest *F*_IS_ was estimated in ARG (0.051) and the highest, excluding the island-isolated MNT_I, was reported in GIR (0.174). The *MAF* averaged per geographical group followed the trend of *H*_E_, showing the highest value in Italy group (0.308) and the lowest in the Asia group (0.261). RCC highlighted the highest breed value (0.328), while CAN the lowest (0.229).

**Table 1:** Dataset composition.

CODE	Breed	Name	*n*_raw_	*n*_final_	GEO	CODE	Breed	Name	*n*_raw_	*n*_final_	GEO
Italy	ARG	Argentata dell'Etna*	48	30	Italy	Alpine arch	ALP	Camosciata delle Alpi*	143	30	Italy
Italy	ASP	Capra dell’Aspromonte*	24	24	Italy	Alpine arch	BIO	Bionda dell’Adamello*	24	24	Italy
Italy	BIA	Bianca Monticellana*	24	24	Italy	Alpine arch	LIV	Capra di Livo-Lariana*	24	23	Italy
Italy	CAP	Capestrina*	24	24	Italy	Alpine arch	NVE	Nera di Verzasca*	19	19	Italy
Italy	FAC	Facciuta della Valnerina*	24	24	Italy	Alpine arch	ORO	Orobica*	23	23	Italy
Italy	FUL	Fulva del Lazio*	22	22	Italy	Alpine arch	RCC	Roccaverano*	28	28	Italy
Italy	GAR	Garganica*	40	30	Italy	Alpine arch	SAA	Saanen*	44	30	Italy
Italy	GCI	Grigia Ciociara*	43	30	Italy	Alpine arch	VAL	Valdostana*	24	24	Italy
Italy	GIR	Girgentana*	59	30	Italy	Alpine arch	VLS	Vallesana*	24	24	Italy
Italy	GRF	Garfagnana*	28	27	Italy	Alpine arch	VPS	Capra della Val Passiria*	24	24	Italy
Italy	JON	Jonica*	16	16	Italy						
Italy	MAL	Maltese*	31	30	Italy	Africa	ABR	Abergelle**	53	30	Ethiopia
Italy	MES	Messinese*	24	24	Italy	Africa	BRK	Barki**	153	30	Egypt
Italy	MNT_I	Montecristo (island)*	24	24	Italy	Africa	GUE	Guera**	25	25	Mali
Italy	MON	Capra di Montefalcone*	24	24	Italy	Africa	GUM	Gumez**	41	30	Ethiopia
Italy	M×S	Maltese x Sarda*	36	30	Italy	Africa	NBN	Nubian**	84	30	Egypt
Italy	NIC	Nicastrese*	24	24	Italy						
Italy	RME	Rossa Mediterranea*	78	30	Italy	Asia	BEZ	Bezoar**	7	7	Wild
Italy	SAR	Sarda*	33	30	Italy	Asia	JAT	Jattan**	24	24	Pakistan
Italy	TER	Capra di Teramo*	43	30	Italy	Asia	KAC	Kachan**	24	24	Pakistan
						Asia	KIL	Kil**	25	25	Turkey
Europe	CRS	Corse**	30	30	Corsica	Asia	KLS	Kilis**	40	30	Turkey
Europe	FSS	Fosses**	26	26	France	Asia	PAT	Pateri**	37	30	Pakistan
Europe	LNR	Landrace**	120	30	Denmark	Asia	TAP	Tapri**	24	24	Pakistan
Europe	MLG	Malaguena**	42	30	Spain						
Europe	PTV	Poitevine**	29	29	France	Brazil	CAN	Caninde**	31	30	Brazil
Europe	PYR	Pyrenean**	27	27	France						

*CODE* geographic group, *Breed* breed’s acronym, *Name* breed’s full name, *n*_*raw*_ the raw breed’s size, *n*_*final*_ reduced size after data management and quality control, *GEO* geographic area of breeding.

*Reference: Cortellari et al.^20^.

**Reference: Stella et al.^18^.

### Genetic relationships

**Figure 1:**
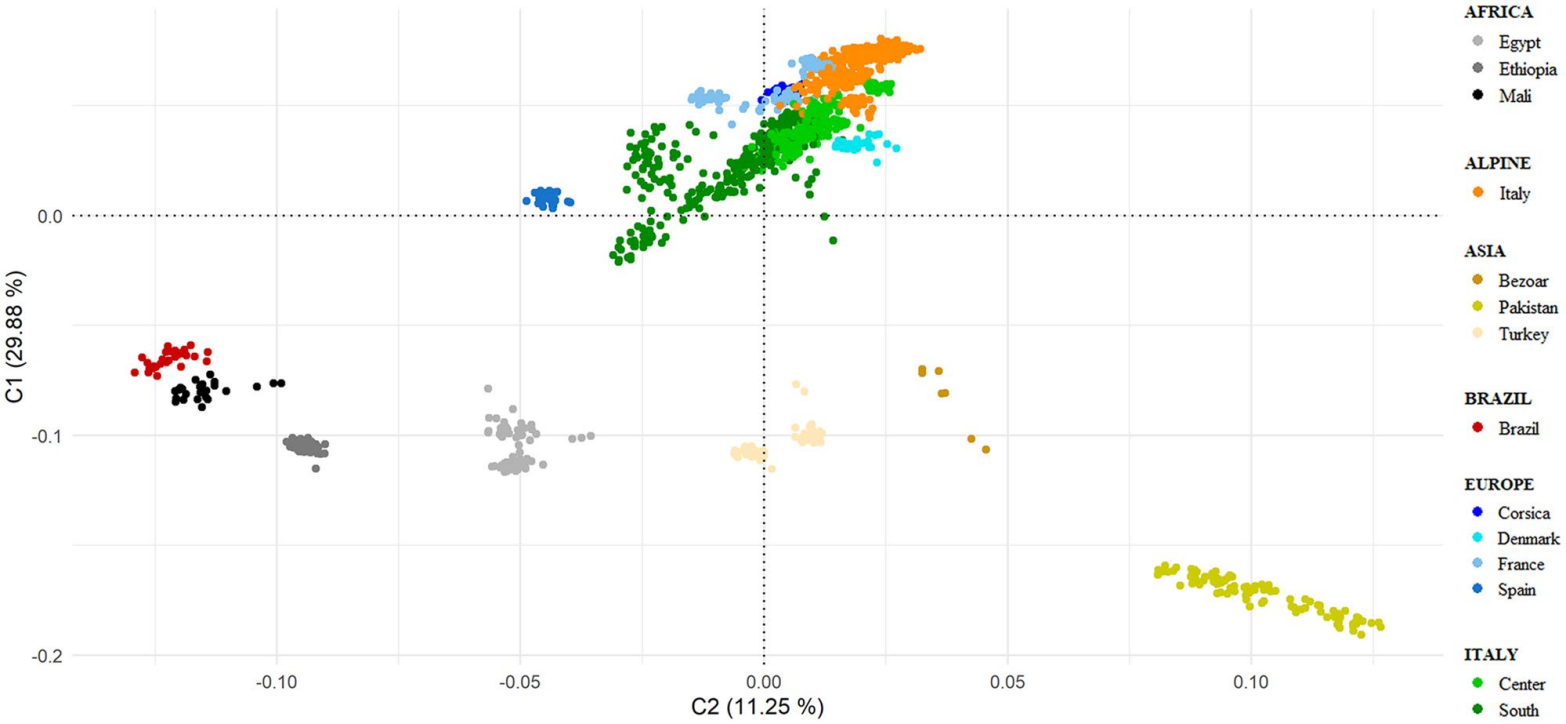
Multidimensional scaling (MDS) plot according to C1 (29.88%) and C2 (11.25%) components. The geographical clusters are represented by different shades of a color. See Table 1 for a full definition of the dataset.

A representation of genetic relationships within- and between-breeds comes from multidimensional scaling analysis. Two different graphs of the same MDS analysis were generated: the first (Fig. 1) shows the breeds grouped according to their geographic breeding area as reported in Table 1, and the second (see Supplementary Fig. S1) represents all breeds separately. In Fig. 1, the first component (C1 = 29.88%) clearly separated the European and Italian breeds from the rest of the dataset: African and Asian breeds clustered according to their different breeding location, while European, Alpine, and Italian breeds partially overlapped and showed a north–south gradient of variation. Focusing on this cluster, the spatial breeds grouping highlighted the effective geographic distribution within each country and between countries, with the Southern, Center, and Northern (Alpine cluster) Italian breeds extending along a line. The second component (C2 = 11.25%) reported a partial overlapping between Spanish and Egyptian breeds, as well as between Turkish, Bezoar and the European macro cluster (Alpine, Italian, and French breeds). Considering both C1 and C2 components, the Spanish MLG breed and PYR from France slightly detached from the European macro cluster (see Supplementary Fig. S1). The Neighbor-Net based on pairwise Reynolds’ genetic distances also shows distinct clustering according to the different geographic distribution of breeds (see Supplementary Fig. S2). In particular, a phylogenetic divergence between European and non-European breeds was highlighted, with the MAL and MLG breeds at the basis of the separation. The Italian breeds highlighted a complex interweaving of nodes and positioned at the center of the Net. Alpine and French breeds clustered showing a certain degree of distinction from other European breeds. The Brazilian breed (CAN), MNT_I and breeds from Asia showed the highest degree of divergence with respect to the analyzed goat breeds.

### Heterozygosity-rich regions

**Figure 2:**
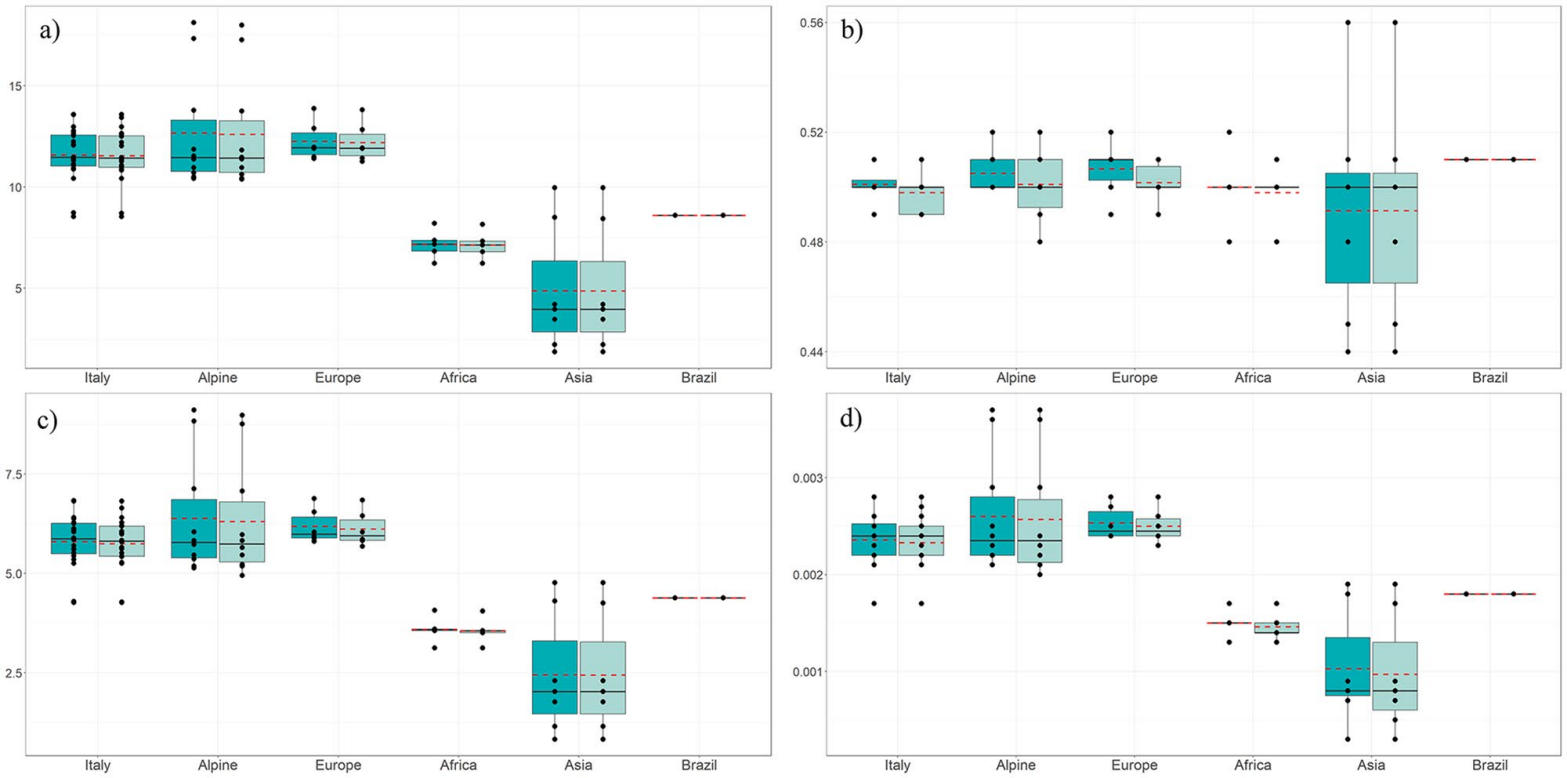
Boxplots of the Heterozigosity-rich regions (HRR) indices aggregated by geographic area. (**a**) *N*_HRR_ = average number of HRR per breed, (**b**) *L*_HRR_ = average length of HRR per breed (in Mbp), (**c**) *S*_HRR_ = mean genome length covered by HRR segments (in Mbp), (**d**) *D*_HRR_ = average coefficient of diversity. The different colors indicate the two approaches, CR (blue) and SW (light blue). Each boxplot extends from the 25th to the 75th percentile and shows the average (red dashed line) and the median (black horizontal line) group value. The black dots represent the single-breed results.

The HRR analysis was performed using two methods of investigation (CR and SW) and reported a total of 13,612 HRR for CR approach and 13,558 HRR for SW approach. Individuals reporting no HRR belonged to Asian breeds (3 animals for KAC, 1 animal for PAT, and 1 animal for TAP). Table 2 shows the descriptive statistics of HRR per breed and geographic cluster. Pearson correlation coefficients were calculated between average values (over the 49 breeds) of the four parameters (*N*_HRR_, *L*_HRR,_ *S*_HRR_ and *D*_HRR_) obtained with the two methods (CR and SW), reporting an *r* > 0.999 for *N*_HRR_, *S*_HRR_ and *D*_HRR_ and an *r* = 0.947 for *L*_HRR_. Based on the high overlapping of the HRR results and high correlation coefficients, we here referred only to the results of the CR approach that reported the highest total number of HRR. Figure 2 graphically presented the results for each parameter per breed and geographical group. The Alpine group had the highest average values within the dataset (*AN*_HRR_ = 12.66, *AL*_HRR_ = 0.51, *AS*_HRR_ = 6.38 and *AD*_HRR_ = 0.0026), with ALP and SAA showing high values above the average both within the group and in the entire dataset. Similar averages were exhibited by the breeds of the Europe group, with MLG breed showing the highest values. The Italy group had mean values of the HRR parameters close to those of Alpine and Europe and showed MON and M×S with the highest values, while MNT_I and GIR were the ones with the lowest. The rest of the goat breeds analyzed showed a fair level of rich heterozygosity and values not far from the group means (*AN*_HRR_ = 11.59, *AL*_HRR_ = 0.50, *AS*_HRR_ = 5.80 and *AD*_HRR_ = 0.0024). Both African and Asian breeds reported low values for all parameters. Within the first group, breeds showed comparable average values, while in the second group, KIL and KLS breeds from Turkey differentiated and showed values above the average.

**Table 2:** Descriptive statistics for Heterozigosity-rich regions (HRR) by consecutive runs (CR) and sliding window (SW) methods.

CODE	Breed	Variable	CR	SW	Breed	Variable	CR	SW	Breed	Variable	CR	SW
			Mean ± s.d	Mean ± s.d			Mean ± s.d	Mean ± s.d			Mean ± s.d	Mean ± s.d
Italy	ARG	*N*_HRR_	11.37 ± 2.93	11.33 ± 2.87	GCI	*N*_HRR_	11.07 ± 3.61	11.07 ± 3.61	MON	*N*_HRR_	13.58 ± 4.86	13.58 ± 4.86
		*L*_HRR_	0.50 ± 0.03	0.50 ± 0.03		*L*_HRR_	0.50 ± 0.03	0.50 ± 0.03		*L*_HRR_	0.50 ± 0.03	0.50 ± 0.03
		*S*_HRR_	5.71 ± 1.66	5.66 ± 1.55		*S*_HRR_	5.51 ± 1.80	5.51 ± 1.80		*S*_HRR_	6.82 ± 2.58	6.82 ± 2.58
		*D*_HRR_	0.0023 ± 0.0007	0.0023 ± 0.0006		*D*_HRR_	0.0022 ± 0.0007	0.0022 ± 0.0007		*D*_HRR_	0.0028 ± 0.0011	0.0028 ± 0.0011
	ASP	*N*_HRR_	10.92 ± 2.76	10.88 ± 2.85	GIR	*N*_HRR_	8.73 ± 3.12	8.70 ± 3.11	M×S	*N*_HRR_	13.57 ± 3.21	13.43 ± 3.28
		*L*_HRR_	0.50 ± 0.04	0.50 ± 0.04		*L*_HRR_	0.49 ± 0.05	0.49 ± 0.05		*L*_HRR_	0.51 ± 0.05	0.50 ± 0.03
		*S*_HRR_	5.44 ± 1.45	5.42 ± 1.47		*S*_HRR_	4.30 ± 1.57	4.28 ± 1.57		*S*_HRR_	6.84 ± 1.57	6.65 ± 1.60
		*D*_HRR_	0.0022 ± 0.0006	0.0022 ± 0.0006		*D*_HRR_	0.0017 ± 0.0006	0.0017 ± 0.0006		*D*_HRR_	0.0028 ± 0.0006	0.0027 ± 0.0007
	BIA	*N*_HRR_	10.88 ± 3.80	10.83 ± 3.80	GRF	*N*_HRR_	12.07 ± 3.34	12.04 ± 3.32	NIC	*N*_HRR_	10.42 ± 3.76	10.42 ± 3.76
		*L*_HRR_	0.49 ± 0.03	0.49 ± 0.03		*L*_HRR_	0.51 ± 0.03	0.50 ± 0.03		*L*_HRR_	0.50 ± 0.05	0.50 ± 0.05
		*S*_HRR_	5.35 ± 1.87	5.28 ± 1.84		*S*_HRR_	6.12 ± 1.83	6.06 ± 1.79		*S*_HRR_	5.25 ± 1.91	5.25 ± 1.91
		*D*_HRR_	0.0022 ± 0.0008	0.0021 ± 0.0008		*D*_HRR_	0.0025 ± 0.0007	0.0025 ± 0.0007		*D*_HRR_	0.0021 ± 0.0008	0.0021 ± 0.0008
	CAP	*N*_HRR_	12.25 ± 3.34	12.21 ± 3.30	JON	*N*_HRR_	11.13 ± 4.32	11.00 ± 4.41	RME	*N*_HRR_	11.47 ± 2.80	11.40 ± 2.76
		*L*_HRR_	0.50 ± 0.04	0.49 ± 0.03		*L*_HRR_	0.51 ± 0.05	0.49 ± 0.04		*L*_HRR_	0.51 ± 0.04	0.51 ± 0.04
		*S*_HRR_	6.05 ± 1.58	6.00 ± 1.51		*S*_HRR_	5.61 ± 2.12	5.43 ± 2.21		*S*_HRR_	5.89 ± 1.55	5.80 ± 1.49
		*D*_HRR_	0.0025 ± 0.0006	0.0024 ± 0.0006		*D*_HRR_	0.0023 ± 0.0009	0.0022 ± 0.0009		*D*_HRR_	0.0024 ± 0.0006	0.0024 ± 0.0006
	FAC	*N*_HRR_	12.54 ± 3.97	12.50 ± 3.95	MAL	*N*_HRR_	12.77 ± 4.05	12.60 ± 4.12	SAR	*N*_HRR_	12.10 ± 2.89	12.03 ± 2.89
		*L*_HRR_	0.50 ± 0.04	0.50 ± 0.04		*L*_HRR_	0.50 ± 0.04	0.49 ± 0.03		*L*_HRR_	0.50 ± 0.04	0.50 ± 0.03
		*S*_HRR_	6.25 ± 2.12	6.20 ± 2.08		*S*_HRR_	6.38 ± 2.08	6.190 ± 2.147		*S*_HRR_	6.10 ± 1.56	6.02 ± 1.53
		*D*_HRR_	0.0025 ± 0.0009	0.0025 ± 0.0009		*D*_HRR_	0.0026 ± 0.0008	0.0025 ± 0.0009		*D*_HRR_	0.0025 ± 0.0006	0.0024 ± 0.0006
	FUL	*N*_HRR_	12.64 ± 3.99	12.64 ± 3.99	MES	*N*_HRR_	11.46 ± 2.52	11.46 ± 2.52	TER	*N*_HRR_	11.30 ± 2.29	11.27 ± 2.29
		*L*_HRR_	0.50 ± 0.04	0.50 ± 0.04		*L*_HRR_	0.49 ± 0.04	0.49 ± 0.04		*L*_HRR_	0.51 ± 0.05	0.51 ± 0.05
		*S*_HRR_	6.28 ± 1.83	6.28 ± 1.83		*S*_HRR_	5.62 ± 1.44	5.62 ± 1.44		*S*_HRR_	5.85 ± 1.31	5.83 ± 1.31
		*D*_HRR_	0.0026 ± 0.0007	0.0026 ± 0.0007		*D*_HRR_	0.0023 ± 0.0006	0.0023 ± 0.0006		*D*_HRR_	0.0024 ± 0.0005	0.0024 ± 0.0005
	GAR	*N*_HRR_	12.97 ± 4.75	12.97 ± 4.75	MNT_I	*N*_HRR_	8.54 ± 2.55	8.54 ± 2.55	Average	*AN*_HRR_	11.59 ± 1.35	11.55 ± 1.34
		*L*_HRR_	0.50 ± 0.03	0.50 ± 0.03		*L*_HRR_	0.50 ± 0.03	0.50 ± 0.03		*AL*_HRR_	0.50 ± 0.01	0.50 ± 0.01
		*S*_HRR_	6.41 ± 2.29	6.41 ± 2.29		*S*_HRR_	4.27 ± 1.28	4.27 ± 1.28		*AS*_HRR_	5.80 ± 0.69	5.75 ± 0.67
		*D*_HRR_	0.0026 ± 0.0009	0.0026 ± 0.0009		*D*_HRR_	0.0017 ± 0.0005	0.0017 ± 0.0005		*AD*_HRR_	0.0024 ± 0.0003	0.0023 ± 0.0003
Alpine arch	ALP	*N*_HRR_	17.33 ± 3.88	17.27 ± 3.92	ORO	*N*_HRR_	10.96 ± 3.42	10.96 ± 3.42	VAL	*N*_HRR_	10.42 ± 3.08	10.42 ± 3.08
		*L*_HRR_	0.51 ± 0.03	0.51 ± 0.03		*L*_HRR_	0.50 ± 0.03	0.50 ± 0.03		*L*_HRR_	0.50 ± 0.04	0.50 ± 0.04
		*S*_HRR_	8.83 ± 1.97	8.76 ± 2.04		*S*_HRR_	5.46 ± 1.77	5.46 ± 1.77		*S*_HRR_	5.19 ± 1.49	5.19 ± 1.49
		*D*_HRR_	0.0036 ± 0.0008	0.0036 ± 0.0008		*D*_HRR_	0.0022 ± 0.0007	0.0022 ± 0.0007		*D*_HRR_	0.0021 ± 0.0006	0.0021 ± 0.0006
	BIO	*N*_HRR_	10.71 ± 2.51	10.63 ± 2.45	RCC	*N*_HRR_	13.79 ± 3.89	13.75 ± 3.86	VLS	*N*_HRR_	10.50 ± 3.39	10.38 ± 3.50
		*L*_HRR_	0.50 ± 0.04	0.49 ± 0.04		*L*_HRR_	0.52 ± 0.03	0.52 ± 0.04		*L*_HRR_	0.50 ± 0.07	0.48 ± 0.03
		*S*_HRR_	5.36 ± 1.45	5.23 ± 1.31		*S*_HRR_	7.13 ± 1.99	7.07 ± 1.94		*S*_HRR_	5.14 ± 1.57	4.95 ± 1.66
		*D*_HRR_	0.0022 ± 0.0006	0.0021 ± 0.0005		*D*_HRR_	0.0029 ± 0.0008	0.0029 ± 0.0008		*D*_HRR_	0.0021 ± 0.0006	0.0020 ± 0.0007
	LIV	*N*_HRR_	11.87 ± 3.90	11.83 ± 3.92	SAA	*N*_HRR_	18.13 ± 4.96	18.00 ± 5.05	VPS	*N*_HRR_	11.38 ± 3.49	11.38 ± 3.49
		*L*_HRR_	0.51 ± 0.06	0.51 ± 0.04		*L*_HRR_	0.50 ± 0.03	0.50 ± 0.03		*L*_HRR_	0.51 ± 0.04	0.51 ± 0.04
		*S*_HRR_	6.05 ± 2.00	5.98 ± 1.97		*S*_HRR_	9.12 ± 2.56	8.98 ± 2.62		*S*_HRR_	5.83 ± 1.89	5.83 ± 1.89
		*D*_HRR_	0.0025 ± 0.0008	0.0024 ± 0.0008		*D*_HRR_	0.0037 ± 0.0010	0.0037 ± 0.0011		*D*_HRR_	0.0024 ± 0.0008	0.0024 ± 0.0008
	NVE	*N*_HRR_	11.53 ± 2.84	11.47 ± 2.93					Average	*AN*_HRR_	12.66 ± 2.85	12.61 ± 2.83
		*L*_HRR_	0.50 ± 0.04	0.49 ± 0.03						*AL*_HRR_	0.51 ± 0.01	0.50 ± 0.01
		*S*_HRR_	5.73 ± 1.30	5.65 ± 1.43						*AS*_HRR_	6.38 ± 1.48	6.31 ± 1.47
		*D*_HRR_	0.0023 ± 0.0005	0.0023 ± 0.0006						*AD*_HRR_	0.0026 ± 0.0006	0.0026 ± 0.0006
Europe	CRS	*N*_HRR_	11.97 ± 3.10	11.93 ± 3.03	MLG	*N*_HRR_	12.90 ± 3.47	12.83 ± 3.46	PYR	*N*_HRR_	11.41 ± 3.55	11.26 ± 3.50
		*L*_HRR_	0.49 ± 0.03	0.49 ± 0.03		*L*_HRR_	0.51 ± 0.04	0.50 ± 0.03		*L*_HRR_	0.51 ± 0.04	0.50 ± 0.04
		*S*_HRR_	5.88 ± 1.54	5.83 ± 1.41		*S*_HRR_	6.55 ± 1.88	6.45 ± 1.85		*S*_HRR_	5.81 ± 1.98	5.68 ± 1.92
		*D*_HRR_	0.0024 ± 0.0006	0.0024 ± 0.0006		*D*_HRR_	0.0027 ± 0.0008	0.0026 ± 0.0008		*D*_HRR_	0.0024 ± 0.0008	0.0023 ± 0.0008
	FSS	*N*_HRR_	13.88 ± 3.76	13.81 ± 3.78	PTV	*N*_HRR_	11.90 ± 2.99	11.90 ± 2.99	Average	*AN*_HRR_	12.26 ± 0.95	12.19 ± 0.96
		*L*_HRR_	0.50 ± 0.03	0.50 ± 0.03		*L*_HRR_	0.51 ± 0.04	0.51 ± 0.04		*AL*_HRR_	0.51 ± 0.01	0.50 ± 0.01
		*S*_HRR_	6.89 ± 1.89	6.84 ± 1.91		*S*_HRR_	6.04 ± 1.54	6.04 ± 1.54		*AS*_HRR_	6.18 ± 0.44	6.12 ± 0.44
		*D*_HRR_	0.0028 ± 0.0008	0.0028 ± 0.0008		*D*_HRR_	0.0025 ± 0.0006	0.0025 ± 0.0006		*AD*_HRR_	0.0025 ± 0.0002	0.0025 ± 0.0002
	LNR	*N*_HRR_	11.50 ± 4.10	11.43 ± 4.13								
		*L*_HRR_	0.52 ± 0.04	0.51 ± 0.04								
		*S*_HRR_	5.94 ± 2.18	5.86 ± 2.21								
		*D*_HRR_	0.0024 ± 0.0009	0.0024 ± 0.0009								
Africa	ABR	*N*_HRR_	6.83 ± 2.25	6.80 ± 2.20	GUE	*N*_HRR_	8.20 ± 3.49	8.16 ± 3.40	NBN	*N*_HRR_	6.23 ± 3.00	6.23 ± 3.00
		*L*_HRR_	0.52 ± 0.06	0.51 ± 0.05		*L*_HRR_	0.50 ± 0.03	0.50 ± 0.03		*L*_HRR_	0.50 ± 0.04	0.50 ± 0.04
		*S*_HRR_	3.56 ± 1.32	3.50 ± 1.22		*S*_HRR_	4.07 ± 1.71	4.05 ± 1.67		*S*_HRR_	3.12 ± 1.59	3.12 ± 1.59
		*D*_HRR_	0.0015 ± 0.0005	0.0014 ± 0.0005		*D*_HRR_	0.0017 ± 0.0007	0.0017 ± 0.0007		*D*_HRR_	0.0013 ± 0.0007	0.0013 ± 0.0007
	BRK	*N*_HRR_	7.37 ± 3.88	7.33 ± 3.84	GUM	*N*_HRR_	7.17 ± 2.79	7.13 ± 2.79	Average	*AN*_HRR_	7.16 ± 0.72	7.13 ± 0.71
		*L*_HRR_	0.48 ± 0.05	0.48 ± 0.05		*L*_HRR_	0.50 ± 0.04	0.50 ± 0.03		*AL*_HRR_	0.51 ± 0.01	0.50 ± 0.01
		*S*_HRR_	3.59 ± 1.97	3.56 ± 1.94		*S*_HRR_	3.60 ± 1.44	3.56 ± 1.43		*AS*_HRR_	3.59 ± 0.34	3.56 ± 0.33
		*D*_HRR_	0.0015 ± 0.0008	0.0014 ± 0.0008		*D*_HRR_	0.0015 ± 0.0006	0.0015 ± 0.0009		*AD*_HRR_	0.0015 ± 0.0001	0.0015 ± 0.0002
Asia	BEZ	*N*_HRR_	1.86 ± 0.90	1.86 ± 0.90	KIL	*N*_HRR_	9.96 ± 3.81	9.96 ± 3.81	PAT	*N*_HRR_	4.20 ± 2.12	4.20 ± 2.12
		*L*_HRR_	0.44 ± 0.03	0.44 ± 0.03		*L*_HRR_	0.48 ± 0.02	0.48 ± 0.02		*L*_HRR_	0.56 ± 0.17	0.56 ± 0.17
		*S*_HRR_	0.83 ± 0.42	0.83 ± 0.42		*S*_HRR_	4.77 ± 1.86	4.77 ± 1.86		*S*_HRR_	2.30 ± 1.05	2.30 ± 1.05
		*D*_HRR_	0.0003 ± 0.0002	0.0003 ± 0.0002		*D*_HRR_	0.0019 ± 0.0007	0.0019 ± 0.0008		*D*_HRR_	0.0009 ± 0.0004	0.0009 ± 0.0004
	JAT	*N*_HRR_	3.46 ± 1.64	3.46 ± 1.64	KLS	*N*_HRR_	8.50 ± 2.43	8.43 ± 2.43	TAP	*N*_HRR_	3.96 ± 2.05	3.96 ± 2.05
		*L*_HRR_	0.50 ± 0.05	0.50 ± 0.05		*L*_HRR_	0.51 ± 0.03	0.51 ± 0.03		*L*_HRR_	0.50 ± 0.13	0.50 ± 0.13
		*S*_HRR_	1.77 ± 0.91	1.77 ± 0.91		*S*_HRR_	4.31 ± 1.22	4.25 ± 1.22		*S*_HRR_	2.03 ± 1.04	2.03 ± 1.04
		*D*_HRR_	0.0007 ± 0.0004	0.0007 ± 0.0004		*D*_HRR_	0.0018 ± 0.0005	0.0017 ± 0.0005		*D*_HRR_	0.0008 ± 0.0004	0.0008 ± 0.0004
	KAC	*N*_HRR_	2.21 ± 1.67	2.21 ± 1.67					Average	*AN*_HRR_	4.88 ± 3.12	4.87 ± 3.11
		*L*_HRR_	0.45 ± 0.18	0.45 ± 0.18						*AL*_HRR_	0.49 ± 0.04	0.49 ± 0.04
		*S*_HRR_	1.15 ± 0.90	1.15 ± 0.90						*AS*_HRR_	2.45 ± 1.52	2.44 ± 1.51
		*D*_HRR_	0.0008 ± 0.0004	0.0005 ± 0.0004						*AD*_HRR_	0.0010 ± 0.0006	0.0010 ± 0.0006
Brazil	CAN	*N*_HRR_	8.60 ± 2.97	8.60 ± 2.97								
		*L*_HRR_	0.51 ± 0.05	0.51 ± 0.05								
		*S*_HRR_	4.39 ± 1.53	4.39 ± 1.53								
		*D*_HRR_	0.0018 ± 0.0006	0.0018 ± 0.0006								

Averages per geographic group are also tabulated (*AN*_HRR_, *AL*_HRR_, *AS*_HRR_ and *AD*_HRR_).

*CODE* geographical cluster, *Breed* breed’s acronym, *N*_*HRR*_ average HRR number, *L*_*HRR*_ average length of HRR (in Mbp), *S*_*HRR*_ mean genome length covered by HRR segments (in Mbp), *D*_*HRR*_ mean coefficient of diversity, *s.d.* standard deviation.

### Heterozygosity islands and gene annotation

**Figure 3:**
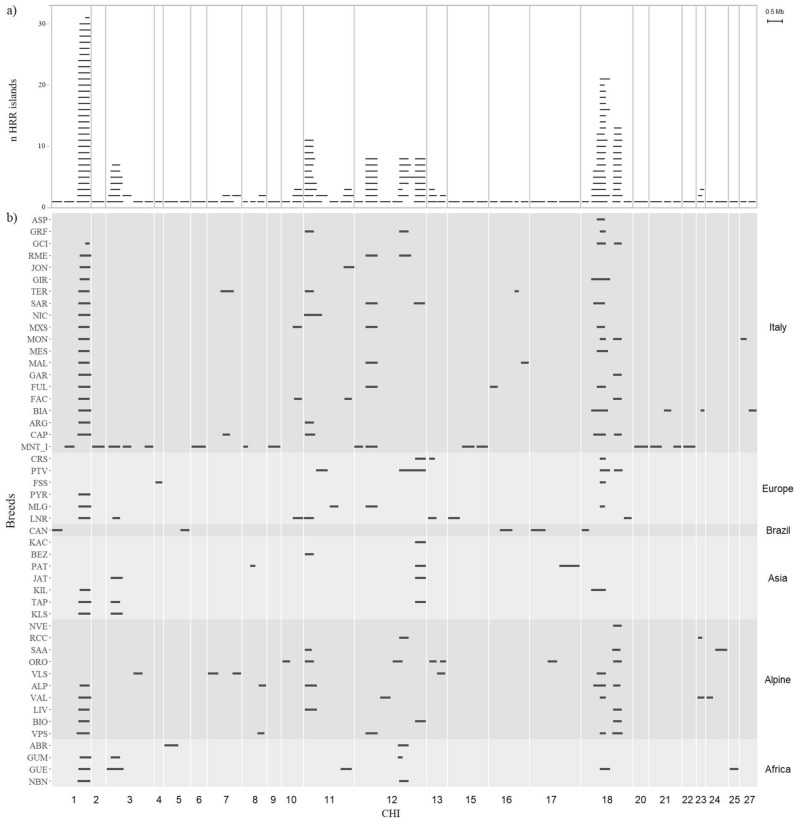
Graphical distribution of Heterozigosity-Rich Regions (HRR) islands per chromosome. Detected HRR in the meta-population (**a**) and per breed (**b**). The gap between consecutive islands within a chromosome and between consecutive chromosomes do not correspond to the real distance in bps.

According to the filter applied for the identification of HRR islands, 1,665 SNPs were considered to form a total of 166 HRR islands (see Supplementary Table S2). The average number of islands on the overall dataset was 3.39 per breed, and the highest number of HRR and islands was found in MNT_I (103 and 16, respectively), while no HRR islands were detected for the BRK breed. To explain the workflow in HRR detection, we report the results of SAA. The 0.1 percentile threshold for cutting off the SNP-in-HRR *p*-values allowed the detection of 164 SNPs for both CR and SW. These markers identified 89 in-breed HRR, but only 21 exceeded the frequency (20% within-breed) and length (minimum 4 SNPs) thresholds, for a final detection of 3 HRR islands. Table 3 reports details of each HRR island including genomic coordinates, breeds, and annotated genes. CHI1, CHI11, CHI12 and CHI18 were the chromosomes harboring the highest number of HRR islands (sum of breeds' HRR islands), reporting a total of 33 (3 unique), 16 (4 unique), 25 (4 unique) and 36 (4 unique) islands, respectively. Several breeds had HRR islands in common. Notably, 31 (including 22 Italian breeds) out of 49 breeds shared an island in CHI1 (CHI1-A from 131.88 to 132.54 Mb). Similarly, a portion of the genome ranging from 36.36 to 40.01 Mb on CHI18, including two HRR islands, was largely shared by several European breeds: 19 breeds had the CHI18-A island in common, and 13 breeds the CHI18-B. Finally, 15 breeds with different geographic distributions reported a common HRR island in CHI12 (CHI12-A from 49.80 to 51.28 Mb). All HRR islands are plotted in Fig. 3, highlighting the chromosome and breeds involved. The gene enrichment performed on genes annotated within HRR islands revealed a total of 74 different processes, mainly biological processes involved into regulation of metabolic processes (CHI12-A), sensor organ development (CHI11-C) and activation of immune response (CHI8-C, CHI16-D) (see Supplementary Table S3). No results were found for CHI1-A shared island.

**Table 3:** Distribution of HRR islands per chromosome.

Chromosome	Code	From	To	Breed	Genes
1	CHI1-A	131,887,965	132,542,315	ALP, ARG, LNR, GUE, KIL, BIO, BIA, MLG, GUM, KLS, LIV, CAP, PYR, NBN, TAP, VAL, FAC, VPS, FUL, GAR, GCI, GIR, JON, MAL, MES, MON, M×S, NIC, RME, SAR, TER	*SLC35G2, STAG1, PCCB*
	CHI1-B	119,276,440	119,721,981	MNT_I	
	CHI1-C	29,543,132	30,017,015	CAN	
2	CHI2-A	61,002,073	61,568,008	MNT_I	
3	CHI3-A	24,833,445	25,571,553	MNT_I, LNR, GUE, JAT, GUM, KLS, TAP	*DMRTA2, FAF1, CDKN2C*
	CHI3-B	68,974,808	69,377,131	VLS	*TGFBR3, BRDT, EPHX4, LOC102178967*
	CHI3-C	61,360,209	61,753,348	MNT_I	*BTBD8, SAMD13, LOC102168228, DNASE2B, RPF1, GNG5, SPATA1, CTBS, SSX2IP*
	CHI3-D	104,971,744	105,344,042	MNT_I	*ASH1L, DAP3, MSTO1, GON4L, YY1AP1, SYT11, RIT1, KHDC4, RXFP4, ARHGEF2*
4	CHI4-A	78,787,857	79,098,602	FSS	
5	CHI5-A	69,680,879	70,284,360	ABR	*ASCL4, RTCB, BPIFC, FBXO7, SYN3, TIMP3*
	CHI5-B	73,464,107	73,868,942	CAN	*MYH9, TXN2, FOXRED2, EIF3D, CACNG2, IFT27*
6	CHI6-A	23,810,805	24,438,981	MNT_I	*PPP3CA*
7	CHI7-A	59,924,105	60,515,404	CAP, TER	*SIL1, CTNNA1, LRRTM2*
	CHI7-B	1,891,473	2,346,215	VLS	*PJA2*
	CHI7-C	71,887,410	72,271,623	VLS	*NSD1, FGFR4, ZNF346, UIMC1, HK3, UNC5A*
8	CHI8-A	74,954,737	75,321,840	ALP, VPS	*AQP3, NOL6, UBE2R2, UBAP2*
	CHI8-B	38,476,907	38,695,165	MNT_I	*RANBP6, KIAA2026, MLANA*
	CHI8-C	39,057,865	39,289,111	PAT	*CD274, PLGRKT, JAK2, INSL6*
9	CHI9-A	50,569,758	51,104,960	MNT_I	*SYNCRIP*
10	CHI10-A	51,511,315	51,976,359	FAC, M×S, LNR	*ADAM10, LOC102184991, MINDY2, SLTM, RNF111, CCNB2, MYO1E*
	CHI10-B	49,756,418	50,111,884	ORO	*TCF12*
11	CHI11-A	37,760,059	38,565,140	ALP, ARG, LNR, BEZ, LIV, CAP, ORO, GRF, SAA, NIC, TER	*CFAP36, PPP4R3B, PNPT1, EFEMP1, CCDC85A*
	CHI11-B	93,996,800	94,603,621	FAC, JON, GUE	*CRB2, DENND1A*
	CHI11-C	60,993,493	61,515,733	PTV	*EHBP1, OTX1, WDPCP*
	CHI11-D	71,126,715	71,509,659	MLG	*BABAM2*
12	CHI12-A	49,805,838	51,283,345	BIO, GRF, CRS, ABR, JAT, ORO, RME, GUM, KAC, RCC, SAR, NBN, PAT, PTV, TAP	*ATP12A, RNF17, CENPJ, PARP4, MPHOSPH8, PSPC1, ZMYM5, GJA3, GJB2, GJB6, CRYL1, IFT88, IL17D, EEF1AKMT1, XPO4, LATS2, SAP18, SKA3, MRPL57, ZDHHC20, MICU2*
	CHI12-B	43,916,255	44,471,339	VPS, FUL, MLG, MAL, MNT_I, M×S, RME, SAR	
	CHI12-C	44,789,048	45,251,150	VAL	
	CHI12-D	36,642,968	37,043,615	MNT_I	
13	CHI13-A	22,047,464	22,437,842	ORO, CRS, LNR	*SKIDA1, MLLT10, DNAJC1*
	CHI13-B	46,470,353	46,869,126	ORO, VLS	*PRNP, PRND, RASSF2, SLC23A2, TMEM230, PCNA*
15	CHI15-A	65,986,490	66,544,220	MNT_I	*GUCY1A2, CWF19L2, C11orf97, FUT4, PIWIL4*
	CHI15-B	72,589,246	73,074,663	MNT_I	*CNTN5*
	CHI15-C	44,309,482	44,822,467	LNR	*PDE3B, CYP2R1, LOC102189885, CRSP-1, CRSP-2, LOC102188877*
16	CHI16-A	33,556,420	33,900,422	FUL	*WDR64, CHML, KMO, FH*
	CHI16-B	54,180,217	54,526,196	MAL	*RABGAP1L*
	CHI16-C	43,284,994	43,460,646	TER	*RERE*
	CHI16-D	35,427,296	35,987,762	CAN	*NME7, LOC108637771, CCDC181, SLC19A2, F5, SELP, SELL, SELE*
17	CHI17-A	54,979,564	55,407,415	ORO	*INPP4B*
	CHI17-B	3,201,875	3,848,371	CAN	*TTC28, PITPNB*
	CHI17-C	60,310,137	61,213,780	PAT	*TMEM184C, PRMT9, ARHGAP10, NR3C2*
18	CHI18-A	36,360,862	37,201,445	ALP, ASP, CRS, GUE, KIL, VAL, BIA, MLG, VLS, CAP, VPS, FUL, FSS, GCI, PTV, GIR, GRF, MES, MON, M×S, SAR	*RIPOR1, CTCF, CARMIL2, ACD, PARD6A, C16orf86, ENKD1, GFOD2, RANBP10, CENPT, TSNAXIP1, THAP11, NUTF2, EDC4, NRN1L, PSKH1, PSMB10, LCAT, SLC12A4, DPEP3, DPEP2, DDX28, DUS2, ESRP2, NFATC3, PLA2G15, SLC7A6, SLC7A6OS, PRMT7, SMPD3, ZFP90*
	CHI18-B	39,567,796	40,014,717	ALP, CAP, BIO, FAC, LIV, GAR, NVE, GCI, ORO, MON, SAA, VPS, PTV	*ZFHX3*
	CHI18-C	40,300,941	40,654,294	LNR	*PKD1L3, IST1, ZNF821, ATXN1L, AP1G1, PHLPP2, MARVELD3*
	CHI18-D	3,788,363	4,113,124	CAN	*LOC102173875, LOC102174148, LOC102176156, BCAR1, BCNT, P97BCNT, TMEM170A, LOC102176721, TMEM231, GABARAPL2*
20	CHI20-A	23,340,984	23,958,224	MNT_I	*IL31RA, DDX4, SLC38A9, PLPP1, MTREX, LOC102190074, DHX29, CCNO, MCIDAS*
21	CHI21-A	51,297,564	51,616,572	BIA	
	CHI21-B	46,698,076	47,218,999	MNT_I	*MIPOL1, FOXA1*
	CHI21-C	64,171,824	64,509,595	MNT_I	*EML1, EVL, DEGS2, YY1, SLC25A29, SLC25A47*
22	CHI22-A	2,644,329	3,178,952	MNT_I	*CMC1, AZI2, ZCWPW2*
23	CHI23-A	36,431,298	36,745,815	RCC, VAL, BIA	*ZFAND3*
24	CHI24-A	43,465,500	44,003,472	SAA	*PTPN2, SEH1L, LDLRAD4, FAM210A, RNMT, MC5R, MC2R*
	CHI24-B	20,161,766	20,444,877	VAL	*KIAA1328*
25	CHI25-A	41,639,292	42,026,061	GUE	*CHST12, EIF3B, SNX8, MRM2, NUDT1, MAD1L1*
27	CHI27-A	36,831,781	37,182,122	BIA	*AGA, NEIL3*
	CHI27-B	12,114,641	12,380,291	MON	*FGFR1, LETM2, NSD3, PLPP5, DDHD2, BAG4, LOC106503669, LSM1, STAR, ASH2L*

For each HRR island, the table reports the island code, spanning range in base pairs (from-to), the list of breeds carrying the island (Breed) and the NCBI name of the annotated genes.

## Discussion

In livestock, selective breeding tends to progressively decrease the diversity and resilience of target breeds^21^. Locally reared breeds still seem to maintain their rustic characteristics thanks to the lower anthropic impact^22^. Goats are well known for being the most suitable livestock species for adapting to harsh environments^18,19^. In particular, local goat breeds represent a way to the sustainability of animal production in marginal areas in both developed and developing countries^18,23^. The domestication process has triggered different evolutionary trends, which over the centuries have led to the development of several breeds with different productive aptitudes. This study aimed to provide a general overview of the relationship among the studied goat breeds and to highlight their HRR patterns as repositories of advantageous gene diversity related to adaptation and resilience processes^6,7^. Identifying a species-specific HRR pattern could provide a clearer view of the mechanisms regulating the genome modelling following anthropogenic selection combined with environmental interaction.

### Genetic diversity indices

The high degree of variability in the dataset reflected the expected biodiversity^19,23,24^ among different breeds. The choice of the dataset, composed of goat breeds claiming a general dairy productive orientation, led to an evident imbalance between the European panorama and the rest of the geographical groups. Therefore, the average diversity values per group need to be considered a function of the notable numerical disparity between European goats and breeds from other parts of the world. Colli et al.^19^ showed a pattern of observed heterozygosity in worldwide goat breeds that matched our results. They highlighted a clear association of this parameter with geography reflecting climatic conditions and breeding management. Therefore, the higher values are probably caused by admixture events between breeds reared in areas with extensive practices (i.e., transhumance in South Italy). The two Sicilian breeds—Argentata dell’Etna and Messinese—represent a known case of occasional admixture linked to the shared breeding area, which impacts on high values of both *H*_*O*_ and *H*_*E*_^25^. Despite their status as cosmopolitan breeds and notoriously more subject to selection, the Saanen and the Camosciata delle Alpi showed relatively high heterozygosity and low molecular inbreeding, proving the proper management of their selective plan. Notably, the feral Montecristo goat resulted in the lowest heterozygosity of the whole dataset, likely reflecting a strong inbreeding due to its geographic isolation. Somenzi et al.^26^ have retraced the evolutionary history of this feral population, suggesting repeated bottleneck events and founder effects that characterized the demographic history of the insular goat.

### Genetic relationships

Genetic relationships analyses pointed out the differences between European and non-European goats, likely as a consequence of gene pool divergence between domesticated breeds and ancestral populations from the regions of the Fertile Crescent^19^. We can also assume that physical barriers, such as long distances and mountain ranges, have marked this divergence. Moreover, bias related to the design of the Illumina chip panel must be considered to explain the clear divergence of the two macro goat clusters^24,27^. The Multidimensional-scaling analysis highlighted the high cohesiveness of the European breeds in their totality (Mediterranean, Central-Europe and Alpine goats) when compared with the rest of the data set. The north–south geographic cline and the greater homogeneity of some breeds such as the Malaguena and the Pyrenean, were particularly evident. More significant variability among the different breeds of the other geographic groups was found (e.g., Pakistani *vs* Turkish goats, Ethiopian *vs* Egyptian goats *vs* Malian goats), probably due to several factors involving the diverse histories of local communities, the different levels of gene flow affecting breeds from different geographic areas (e.g. Pakistani *vs* Turkish goats)^19,28^, the eventual influence of the European genome^29^, as well as the original complexity of the African and Asian goat stock origin^30,31^. The phylogenetic nodes of the Neighbor-Net based on pairwise Reynolds’ genetic distances better represented the genetic relationships among breeds, confirming the genetic closeness of the Europe, Alpine and Italy geographic groups and their divergence from the African and Asiatic strains. The Montecristo goat showed a close relationship to the other insular goat (Corse) and genetic proximity to the Italy group possibly because of the recent inputs of domestic stocks (twentieth century) as already reported in a previous study^26^.

### Heterozygosity-rich regions

HRR are heterozygous genomic regions which are potentially associated with disease resistance, immunity, and adaptation processes. The higher the level of diversity, the better the biological response of species to environmental changes or new diseases. Consecutive Runs and Sliding Window are the two approaches currently used to detect stretches of consecutive homozygosity (ROH) and HRR in livestock species e.g.^3,7,8,32,33^. Although only few studies have focused on identifying HRR islands with both methods of detection, it seems that the use of the Consecutive Runs approach is preferred^3,7,15^. In this study, we investigated both methods, highlighting the overlapping results for HRR identification. Strong correlation between CR and SW was estimated for all the parameters, indicating nearly identical results for all breeds. In a similar study on HRR in pigs, Bordonaro et al.^14^ found a Pearson correlation coefficient higher than 0.96 for all four parameters (*N*_ROH_, *L*_ROH_, *S*_ROH_, *F*_ROH_), confirming the obtaining of overlapping results between the two approaches. As mentioned by Biscarini et al.^6^, the choice of the parameter values is of fundamental importance in detecting ROH/HRR because of its effect on results. The information present in the scientific literature regarding the setting of the detection parameters is not very detailed. According to Mulim et al.^7^, the allowed number of opposite and missing SNP and the gap between two consecutive markers are the factors that mainly affect the detection of runs. In our preliminary screening, we evaluated the number of HRR found by testing several combinations of parameters (the minimum number of SNPs in a run, the minimum length, and the number of missing/opposite markers). The minimum number of SNPs in a HRR (*minSNP*) and the maximum number of SNP with opposite genotype in a window (*maxOppWindow*) were the parameters that most influenced the number of HRR found in SW method, while for the CR method it was the number of opposite genotypes allowed in a HRR (*maxOppRun*)^34^. Selli et al.^8^ conducted a methodological study on several combinations of detection parameters in Sliding Window approach to investigate HRR. They found that a reduction in the minimum number of SNPs in a run (*minSNP*) and in a window (*windowSize*), as well as the number of homozygous and missing SNPs allowed (*maxOppRun* and *maxMissRun*), caused a decrease in HRR found, whereas a reduction in the minimum length of the run (*minLengthBps*) caused an increase in the number of HRR. Similarly, *minSNP* and *windowSize* positively affect the HRR length. In this scenario, it is unclear how different combinations might affect the results, and further analyses are needed^6^. In general, the within-breed occurrence of HRR is lower than that of ROH^2,3,6^. Indeed, we found an average number of HRR per breed lower than 18, while Cortellari et al.^23^ found an average number of ROH of 31.5 in the same Italian breeds data. Other studies on HRR reported an average of 9.5 in semi-feral Chillingham cattle population known to be characterized by a strong inbreeding^4^, less than 9 in the italian Maremmana cattle breed^6^, 121.5 in Pinzgauer cattle breed^2^, 57.8 in commercial turkeys^35^, 52.2 in a local horse breed^3^, ~ 40 in Duroc pig^9^ and 139.6 in sheep^8^. These notable differences may be attributed to the species of interest, to the breeds analysed, and to the different SNP arrays and parameters used for the HRR detection. All the cited studies also found that HRR were much shorter than ROH, confirming our general mean of ~ 500 kb in length and reflecting low coverage of the entire genome (maximum of 6.38 Mb for the Alpine breed group). These results might be related to the absence of missing and opposite SNPs in the detection approach, that usually reduces the size of detected ROH^6^ and, similarly, of HRR. We highlighted a notable difference between the results obtained for European (Italy, Alpine, Europe groups) and non-European clusters (Africa, Asia, Brazil). We assume that this result, also reported by Li et al.^15^ in chinese goats compared to worldwide breeds, could come from ascertainment bias related to the SNP array design based on European goat breeds^24,27^. Worth of note are the outcomes of Maltese × Sarda (M×S) goats. This population represents the only crossbreed sample included in the data set and the high values for all the indices were expected and probably related to the combination of relatively different genomes.

Similarly, Mulim et al.^7^ reported the borderline case of the Montana cattle that combines different genomes due to the crossbreeding between *Bos taurus indicus* and *Bos taurus taurus* and, consequently, showed the highest amount of HRR in their dataset.

### Heterozygosity islands and gene annotation

The identification of recurrent HRR for each breed highlighted wide regions of shared heterozygosity in the chromosomes CHI1, CHI11, CHI12, and CHI18. Williams et al.^4^ observed that heterozygosity-rich regions in the Chillingham cattle genome appear to be not randomly distributed but rather grouped in particular chromosomal locations. Moreover, some of the detected heterozygosity islands had already been recognized as homozygosity islands in previous studies^15,36^. Classifying the same chromosomal regions as both homozygosity-rich and heterozygosity-rich emphasizes the diversity among breeds within the goat species. The different breeding strategies and the different production orientations^15^ rather than the adaptation to different environments have differentially shaped the genome of breeds^14^. CHI1-A (131.88–132.54 Mb) was the most shared island within the whole dataset. *STAG1* and *PCCB* genes, mapped within CHI1-A, are linked to reproduction-related traits and were reported to experience balancing selection in goat breeds, playing a significant role in the domestication of the species^15^. Notably, this HRR island was not present in either Bezoar or Montecristo, the two wild goat populations in the dataset. The second most common HRR island was CHI18-A (36.36–37.20 Mb). It was detected mainly in Italian and Alpines breeds, matched a ROH island found in goats from Europe, Africa and Asia by Bertolini et al.^36^, and it was reported as a possible signature of selection for fiber production with a low number of SNPs supporting this finding^37^. This region harbors genes involved in productive mechanisms in other species, such as the *RIPOR1* gene for feeding intake control in the Nellore cattle breed^38^ or the *PSKH1* gene listed as a candidate gene for mean staple length, live weight, greasy fleece weight, and mean fiber diameter in Merino sheep^39^. The *EDC4* gene was reported to mediate the post-transcriptional regulation of IL-6 cytokine playing an important role in immune response^40^. The nearby island CHI18-B (39.56–40.01 Mb) was detected in 7 Alpine breeds out of 10 and in 5 breeds of the Italy group, allowing us to hypothesize a possible correlation to a well-defined geographic area. This short region harbors the *ZFHX3* gene, also known as *ATBF1*, which has a well-known function in humans as a transcription factor that regulates myogenic and neuronal differentiation, with similar effects on Chinese native goat breeds where its polymorphisms influence animal growth rate^41^. As far as found in literature, a study on 3 Chinese local goat breeds confirmed the association of this gene with development traits^42^. In our results, we did not find this HRR island in any of the six Asian breeds. Bertolini et al.^36^ found two ROH regions in goat chromosome 12 (from 43 to 44 Mb in European breeds and from 50 to 51 Mb in worldwide goats) which overlapped two of our HRR hotspots CHI12-B (43.91–44.47 Mb) and CHI12-A (49.80–51.28 Mb), harboring genes that are related to ectodermal, nervous system, and hearing function (*GJB2* and *GJB6*)^43,44^ as well as gonad development (*SAP18*)^45^. CHI12-A was also reported as a ROH hotspot by Li et al.^15^ in Chinese and other worldwide goat breeds; the authors underlined this region in common with a ROH hotspot in Chinese sheep breeds^46^ and possibly under parallel selection between goats and sheep species before the domestication due to the presence of a series of genes (*GJA3*, *GJB2* and *GJB6*) associated with perception senses, such as sight and hearing essential for surviving in harsh environments (lack of food and presence of wild enemies). These genes were also identified under positive selection for growth (body size, skeletal and embryonic development) in Boar goat^47^ and in Barki goat and sheep^48^. Signatures of selection localized in CHI12-A (49.80–51.28 Mb) were reported in goats by Serranito et al.^22^ which listed a series of annotated genes involved in the adaptation of small ruminants to arid environments (*RNF17*, *ATP12A*, *GJB2*) and high altitude (*ATP12A*, *PARP4*, *ZMYM5*, *PSPC1*, *CENPJ*, *GJB2*). Moreover, the interleukin17D (*IL17D*) gene, spanning from ~ 50.90 to ~ 50.93 Mb and belonging to the *IL17* family of cytokines, was closely associated to host defense and immune response in humans^49,50^. This region perfectly matched the one found in 5 commercial and local goat breeds by Biscarini et al.^51^. CHI11-A (37.76–38.56 Mb) was a shared island among 11 breeds, mainly from the Alpine and Italy groups. This region overlapped the ROH island found by Bertolini et al.^36^, which detected a signature of selection in a previous study^37^. In our dataset, the two cosmopolitan breeds (Saanen and Camosciata delle Alpi) reported this HRR island in which several mapped genes have a role in livestock production. In particular, the *PPP4R3B* gene was associated to thermotolerance in the African N’Dama cattle breed^52^; Berihulay et al.^53^ found this gene involved in gluconeogenesis and lipidic metabolism in Abergelle goats. The *PNPT1* gene was reported implicated into RNA transport in pigs^53^ and Bandur sheep^54^. Previous studies linked the *EFEMP1* gene with traits of interest in livestock species. Zhang et al.^55^ identified this gene as a significant influencer of the oleic acid content in the meat of the Wagyu × Angus cattle breed. Or else, *EFEMP1* was previously identified as a differentiated expressed gene in muscle for residual feed intake in Nelore steers and seems to be regulated by a transcriptional factor (*TCF4*)^56^. The worth of notice are the islands in CHI8, even though they are present only in few breeds: CHI8-A (74.95–75.32 Mb) and CHI8-C (39.05–39.29 Mb) seem to have a strong correlation with immune response. In particular, the *AQP3* gene in the first island was found in a ROH hotspot in Ganxi and Guangfeng goats by Li et al.^15^ who stated its involvement in immunoreactions. The *CD274* and *JAK2* genes in CHI8-C were reported in several biological processes for immune response in our gene enrichment results.

### Heterozygosity islands in feral Montecristo goat

The evolutionary history of the feral Montecristo goat, geographically isolated in the homonymous island, subject to repeated bottleneck phenomena, and in the absence of anthropic management, albeit with a partial introduction of domestic germplasm^26^, represents an outlier case in the analyzed dataset. Moreover, notwithstanding the highest inbreeding coefficient (*F*_IS_) and the lowest heterozygosities (*H*_O_ and* H*_E_) of the whole dataset, Montecristo goat reported the highest number of HRR. Events of balancing selection characterizing this feral goat population might be assumed during the shape of its genomic structure. Despite a high inbreeding, these events may have led to an increase in short segments of heterozygosity, that are strongly associated with fitness and survival traits^2,12^. Indeed, *FAF1* gene in CHI3-A (24.83–25.57 Mb) was highly associated with tolerance to *Theileria* infection in African cattle^57^. On chromosome 3, HRR islands (CHI3-C and CHI3-D) harbored two genes (*SPATA1* and *ASH1L*) connected to reproduction traits in livestock species^58–60^. Moreover, *SYT11* may play an important role in adaptation to different environmental conditions in Kenyan goat breeds^61^, while *ARHGEF2* gene’s function in livestock is still unclear, but may be involved in epithelial barrier permeability affecting host-microbial interactions in the rumen^62^. The HRR island in CHI6-A (23.81–24.43 Mb) was also found as a HRR region in MNT_I by Somenzi et al.^26^, which attributed a reproduction function due to the presence of *PPP3CA* gene associated to fecundity traits and litter size in small ruminants. The HRR islands in CHI8-B (38.47–38.69 Mb), in CHI15-A (65.98–66.54 Mb), and CHI15-B (72.58–73.07 Mb) showed other candidate genes related with immune response traits such as *RANBP6*^63^, *GUCY1A2*^64^, *PIWIL4*^65^, and *CNTN5*^66^. The *IL31RA* gene in CHI20-A (23.34–23.95 Mb) is a cytokine receptors known to be involved in human inflammation and allergic diseases^67^. Moreover, *SLC38A9* gene in the same HRR island was identified as an indicator of heat stress in bovine^68^. Finally, the last gene worth of interest is *EML1* in CHI21-C (64.17–64.51), which plays a role in processes of exocytosis (the process of releasing vesicle content to the extracellular environment) in the molecular pathway of Ab production by B lymphocytes^69^. However, we cannot exclude that the high number of HRR islands found in the feral goat Montecristo derives from the bias linked to the chip design based on the domesticated goat genome.

## Conclusions

This work studied the genetic relationships among goat breeds with dairy aptitude and investigated their continuous heterozygosity patterns. We confirmed a notable divergence between the European lineage and the Asian and African breeds. European goats have shown a clear north–south geographic cline and genetic interconnections probably due to gene flow between geographically close breeds. The investigation of heterozygosity-rich regions highlighted specific portions of the goat genome associated with different functional factors. The distribution of the HRR islands in the goat genome seems to be mainly related to the breeds' geography. A species-specific HRR pattern, possibly shaped by adaptation mechanisms, might provide a clearer view of the mechanisms modelling the genome as a function of anthropic selection. Interestingly, some heterozygosity hotspots showed overlap with ROH islands reported in the literature. This possibly indicates genomic areas targeted by selective breeding which has shaped the goat genome differently in different production contexts. The methodological part of the manuscript gave new insight into the standardization of HRR detection, highlighting the point of overlap between two different detection methods. Further investigation is needed to improve the parameter settings of HRR detection, possibly involving different species and breeds genotyped with different densities of SNP arrays.

## Methods

### Dataset and quality control

A dataset including worldwide goat breeds was generated by merging the Italian Goat Consortium2 (IGC2) and the ADAPTmap repositories described in Cortellari et al.^20^ and Stella et al.^18^, respectively. The breeds involved in the analyses were selected for their milk-production aptitude, excluding those for meat or fiber, and grouped according to their geographic breeding area, resulting in a reduced dataset comprehensive of 1,289 individuals belonging to 49 goat breeds (Table 1). Italian breeds were divided into two different groups (Southern and Center breeds labelled as Italy, while Northern breeds as Alpine arch) due to their isolation and divergent morpho-aptitude factors. The random sampling selection procedure implemented in the bite r package^70^ was used to select a maximum of 30 representative individuals per breed. All animals were genotyped with the Illumina GoatSNP50k BeadChip, including a total of 53,347 SNPs^24^. Chromosomal coordinates of SNPs were referred to the *ARS1* genome assembly (Assembly GCA_001704415.1) using the commands --update-map and --update-chr. The software PLINK ver. 1.9^71^ was used to perform filtering and quality control. After removing the unmapped SNPs and markers located on sexual chromosomes, the additional quality parameters were set as follows: minor allele frequency ≥ 0.05 (--maf 0.05), SNP genotype call rate ≥ 0.95 (--geno 0.05) and individual genotype call rate ≥ 0.80 (--mind 0.20), resulting in 48,544 SNPs and 1287 goats.

### Genetic diversity indices

The software Plink ver. 1.9^71^ was used to estimate the observed and expected heterozygosity (*H*_O_ and *H*_E_), the molecular inbreeding coefficient (*F*_IS_) and calculate the minor allele frequencies (*MAF*) for each breed, using the commands --hardy, --het and --freq respectively. Genetic diversity indices values were averaged per geographical group.

### Genetic relationships

To explore the genetic relationships among breeds, the multidimensional scaling (MDS) analysis was performed based on pairwise identity-by-state (IBS) distances between individuals (--read-genome --cluster --ppc 0.0001 --mds-plot 30 eigvals) using Plink ver. 1.9^71^. In addition, the Neighbor-Net based on pairwise Reynolds’ genetic distances was obtained using software Arlequin ver. 3.5.2.2^72^ and plotted using SplitsTree4 ver. 4.14.8^73^.

### Heterozygosity-rich regions detection

Heterozygosity-rich regions (HRR) were investigated using the R package *detectRUNS* ver. 0.9.6^33^, by two different methods of detection: Sliding Window (SW)^74^ and Consecutive Runs (CR)^75^. The absence of clear guidelines from literature led us to test range of values for the detection parameters^34^. Screening involved several combinations, varying the minimum number of SNPs in a HRR (10–25), the minimum length (10^3^ bp to 10^6^ bp), and the number of missing/opposing markers (0–3). The evaluation criteria to choose the parameters setting were based on (i) avoiding missing or opposite genotype per run; (ii) obtaining a comparable number of HRR between the two methods (SW and CR). The following parameters were finally set: (i) the minimum number of SNPs included in the HRR was 10; (ii) the number of missing or opposite genotypes were set to zero; (iii) the maximum gap between consecutive SNPs was set to 1 Mb; (iv) the minimum HRR length was set to 250 kb. Additional parameters were set for the SW method: v) sliding window of 10 SNPs for HRR; vi) no missing or opposite genotypes were allowed in the window; (vii) the minimum density of one SNP every 100 kb; (viii) the threshold to call a SNP within a HRR was set to 0.05. The HRR pattern per breed was investigated through the average HRR number (*N*_HRR_), the average length of HRR in Mbp (*L*_HRR_) and the mean genome length covered by HRR segments in Mbp (*S*_HRR_). Finally, the total length of the genome covered by HRR per breed was divided by the total autosomal genome length covered by the SNP array (~ 2.4 Gb) to evaluate the coefficient of diversity (*D*_HRR_)^9,14^. In addition, the four descriptive breed-statistics were aggregated by geographic area (*AN*_HRR_, *AL*_HRR_, *AS*_HRR_ and *AD*_HRR_).

### Heterozygosity islands detection and gene annotation

HRR islands per breed were identified comparing the results of SW and CR methods of detection. Based on the standard normal z-score, obtained from all the SNPs-within-HRR incidence per breed and method, *p*-values were calculated and the top 0.1% of SNPs were selected to constitute an island^14,76,77^. Only those markers identified simultaneously with both methods were considered. HRR islands in a breed were considered those regions with frequency ≥ 20% and a minimum of 4 SNPs. The genomic coordinates of HRR islands were examined using the Ensemble browser for the goat genome, according to the assembly *ARS1* (Assembly GCA_001704415.1) to retrieve annotated gene lists. Gene Ontology (GO) and the enrichment analysis of annotated genes were conducted for each island using the open-source Database for Annotation, Visualization, and Integrated Discovery ver. 2021 package^78^. For the GO terms and Kyoto Encyclopaedia of Genes and Genomes (KEGG) pathway analysis, the level of significance for the enriched biological processes was set as *p*-value < 0.05. Corrections for multiple testing were made by applying the Bonferroni test.

### Supplementary Information

Supplementary Information.

## Data Availability

The datasets generated and analyzed during the current study are available in the Mendeley Data repository, https://data.mendeley.com/datasets/hnd59x6gmg/1^20^ and Dryad repository, https://datadryad.org/stash/dataset/doi:10.5061/dryad.v8g21pt^18^.
