# Supplementary material for: Characterization of heterozygosity-rich regions in Italian and worldwide goat breeds
Source: Sci Rep. 2024 Jan 2;14:3. doi: 10.1038/s41598-023-49125-x (PMC10762050; doi:10.1038/s41598-023-49125-x)
Supplement: Supplementary file 1 — Supplementary Information. [file 41598_2023_49125_MOESM1_ESM.pdf]

Characterization of heterozygosity-rich regions in Italian and worldwide goat breeds

Giorgio Chessari, Andrea Criscione, Donata Marletta, Paola Crepaldi, Baldassare Portolano,

Arianna Manunza, Alberto Cesarani, Filippo Biscarini, Salvatore Mastrangelo

Supplementary Table S1 Summary statistics of genetic diversity. Geographic cluster (CODE), breed's acronym (Breed), observed heterozygosity (H_O), expected heterozygosity (H_E), fixation index (F_{IS}) and minus allele frequency (*MAF*) per breed. The mean value of each index per geographical cluster is also reported. See Table 1 for a full definition of the dataset.

**CODE**	**Breed**	H_O		H_E		F_{IS}		*MAF*	
		**mean**	**s.d.**	**mean**	**s.d.**	**mean**	**s.d.**	**mean**	**s.d.**
Italy	ARG	0.416	0.128	0.412	0.100	0.051	0.018	0.326	0.118
	ASP	0.401	0.138	0.403	0.109	0.086	0.057	0.317	0.123
	BIA	0.392	0.140	0.399	0.112	0.106	0.077	0.314	0.125
	CAP	0.405	0.141	0.401	0.109	0.074	0.080	0.315	0.123
	FAC	0.395	0.135	0.407	0.105	0.098	0.099	0.322	0.121
	FUL	0.413	0.139	0.408	0.105	0.057	0.027	0.322	0.120
	GAR	0.407	0.134	0.403	0.107	0.071	0.070	0.316	0.121
	GCI	0.403	0.130	0.408	0.104	0.079	0.056	0.322	0.120
	GIR	0.362	0.157	0.364	0.140	0.174	0.084	0.280	0.140
	GRF	0.397	0.137	0.401	0.111	0.093	0.048	0.315	0.124
	JON	0.415	0.181	0.373	0.133	0.055	0.029	0.288	0.136
	MAL	0.369	0.142	0.384	0.124	0.134	0.102	0.298	0.132
	MES	0.413	0.138	0.406	0.106	0.058	0.024	0.320	0.121
	MNT_I	0.271	0.208	0.263	0.191	0.381	0.016	0.198	0.165
	MON	0.405	0.139	0.405	0.107	0.074	0.097	0.320	0.122
	M×S	0.406	0.127	0.412	0.101	0.072	0.048	0.327	0.118
	NIC	0.394	0.138	0.404	0.108	0.101	0.078	0.318	0.122
	RME	0.396	0.132	0.402	0.109	0.137	0.105	0.316	0.123
	SAR	0.404	0.129	0.408	0.104	0.079	0.043	0.321	0.120
	TER	0.384	0.152	0.382	0.125	0.123	0.080	0.297	0.133
	mean	*0.392*	*0.143*	*0.392*	*0.116*	*0.105*	*0.062*	*0.308*	*0.126*
Europe	CRS	0.400	0.134	0.401	0.109	0.087	0.030	0.315	0.123
	FSS	0.393	0.145	0.391	0.118	0.104	0.080	0.304	0.128
	LNR	0.358	0.144	0.379	0.130	0.184	0.107	0.295	0.135
	MLG	0.415	0.131	0.409	0.104	0.054	0.032	0.324	0.120
	PTV	0.374	0.153	0.372	0.133	0.146	0.047	0.287	0.137
	PYR	0.373	0.148	0.382	0.127	0.149	0.088	0.297	0.133
	mean	*0.386*	*0.143*	*0.389*	*0.120*	*0.121*	*0.064*	*0.304*	*0.129*

**CODE**	**Breed**	H_O		H_E		F_{IS}		*MAF*	
		**mean**	**s.d.**	**mean**	**s.d.**	**mean**	**s.d.**	**mean**	**s.d.**
Alpine arch	ALP	0.409	0.133	0.405	0.107	0.066	0.018	0.319	0.122
	BIO	0.403	0.141	0.401	0.111	0.081	0.039	0.316	0.124
	LIV	0.404	0.146	0.396	0.115	0.078	0.019	0.311	0.127
	NVE	0.391	0.159	0.385	0.126	0.108	0.044	0.300	0.133
	ORO	0.358	0.169	0.352	0.146	0.183	0.046	0.268	0.143
	RCC	0.399	0.126	0.414	0.098	0.089	0.080	0.328	0.116
	SAA	0.413	0.134	0.405	0.106	0.057	0.024	0.319	0.121
	VAL	0.369	0.152	0.376	0.132	0.158	0.095	0.291	0.136
	VLS	0.351	0.167	0.358	0.144	0.199	0.094	0.275	0.142
	VPS	0.400	0.138	0.404	0.109	0.087	0.047	0.319	0.123
	mean	*0.390*	*0.147*	*0.390*	*0.119*	*0.111*	*0.051*	*0.305*	*0.129*
Africa	ABR	0.364	0.162	0.361	0.144	0.169	0.038	0.279	0.143
	BRK	0.399	0.138	0.399	0.114	0.089	0.026	0.314	0.126
	GUE	0.370	0.185	0.344	0.154	0.156	0.051	0.263	0.148
	GUM	0.369	0.158	0.367	0.140	0.157	0.032	0.284	0.140
	NBN	0.355	0.159	0.361	0.143	0.190	0.032	0.277	0.142
	mean	*0.371*	*0.160*	*0.366*	*0.139*	*0.152*	*0.036*	*0.283*	*0.140*
Asia	BEZ	0.276	0.197	0.329	0.166	0.371	0.076	0.252	0.155
	JAT	0.308	0.180	0.313	0.164	0.296	0.128	0.234	0.152
	KAC	0.281	0.204	0.278	0.186	0.359	0.064	0.209	0.163
	KIL	0.401	0.147	0.394	0.117	0.085	0.028	0.309	0.128
	KLS	0.398	0.140	0.396	0.117	0.091	0.044	0.311	0.128
	PAT	0.328	0.177	0.328	0.162	0.251	0.073	0.249	0.152
	TAP	0.331	0.174	0.340	0.158	0.245	0.074	0.260	0.150
	mean	*0.332*	*0.174*	*0.340*	*0.153*	*0.243*	*0.070*	*0.261*	*0.147*
Brazil	CAN	0.333	0.209	0.304	0.174	0.246	0.056	0.229	0.158

Supplementary Figure S1

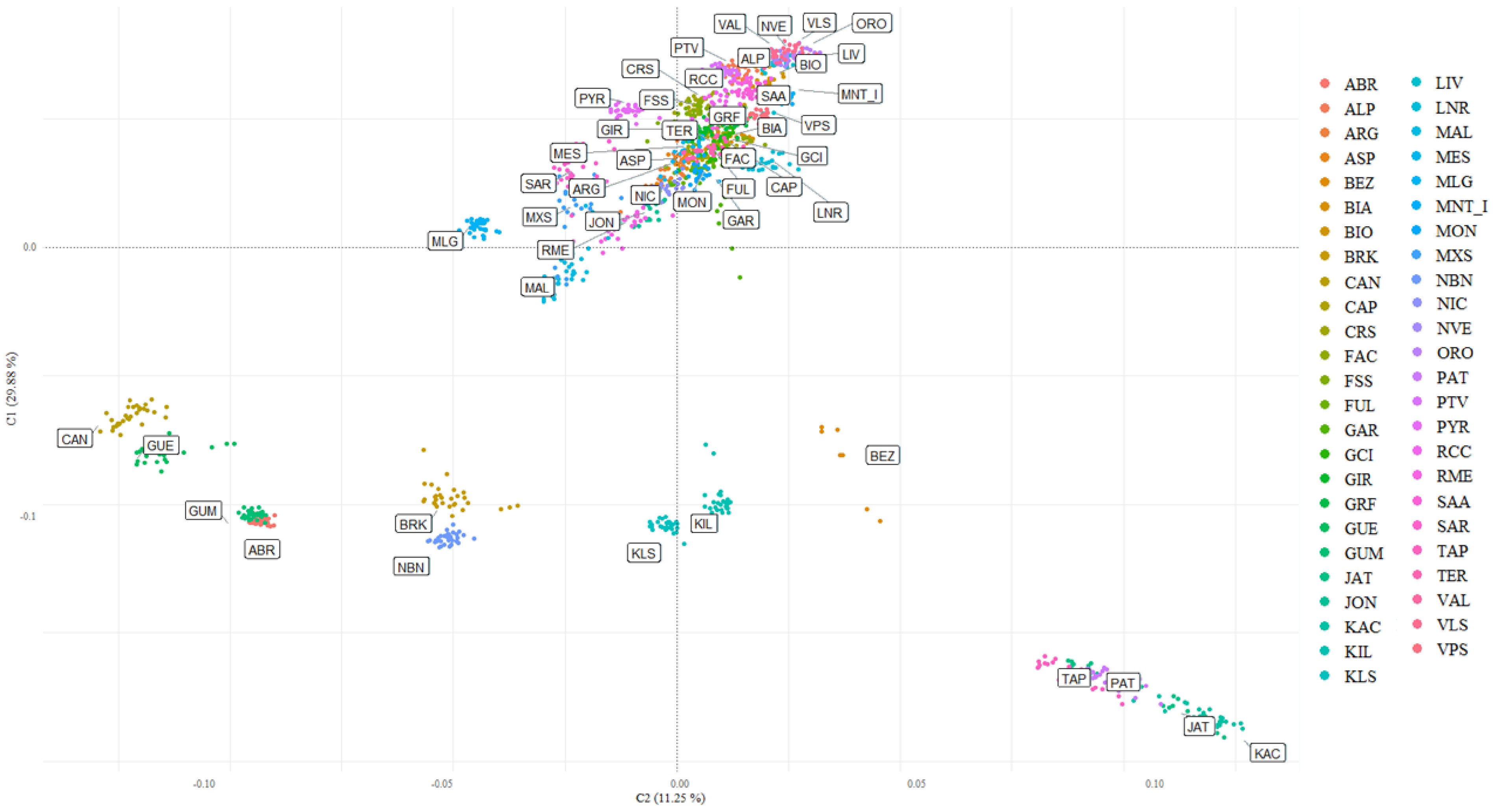

Multidimensional scaling (MDS) plot of the goat dataset, according to the components C1 (29.88%) and C2 (11.25%). Each breed is represented by a different color. See Table 1 for a full definition of the dataset.

Supplementary Figure S2

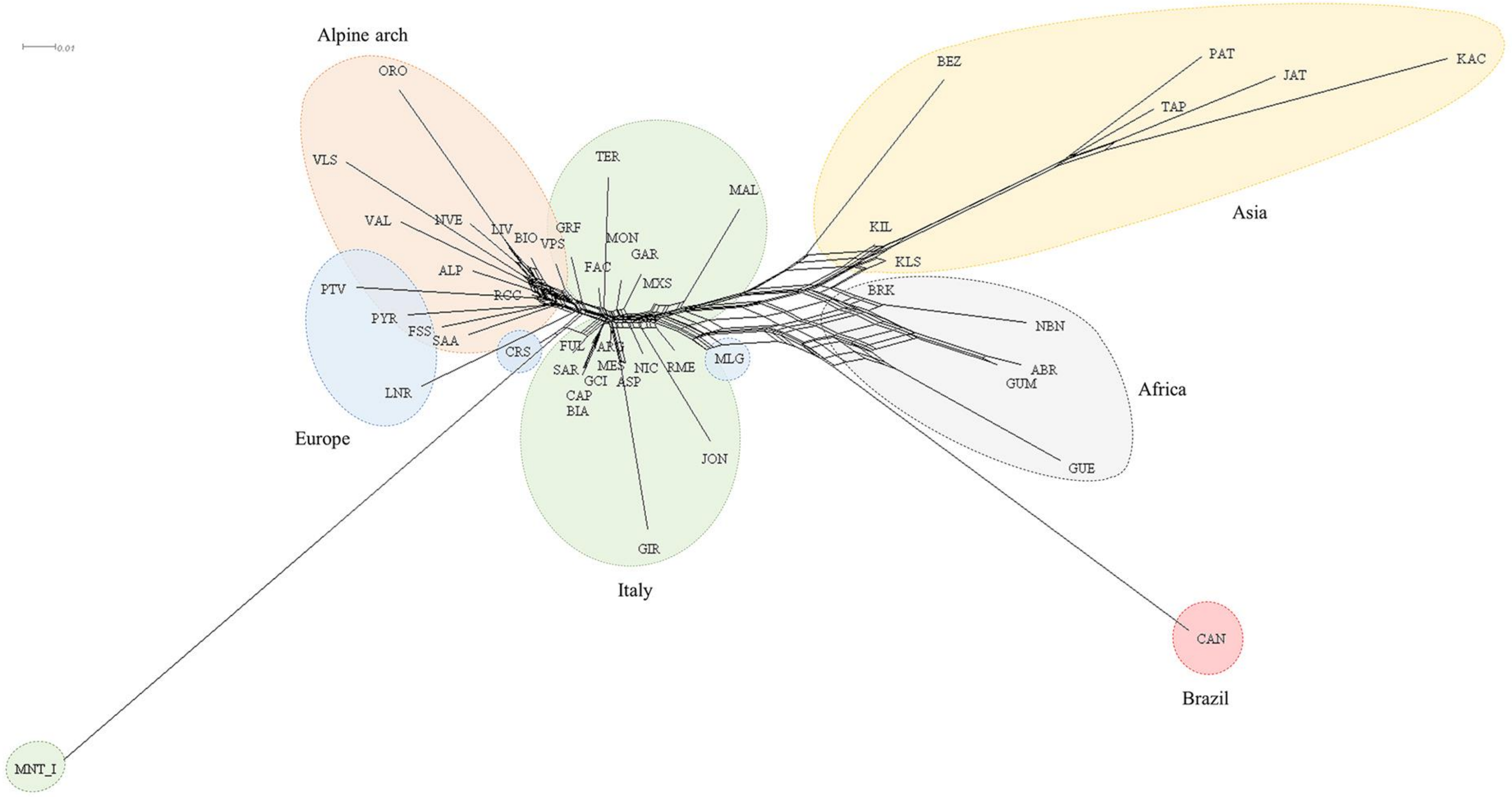

Neighbor-Net based on pairwise Reynold's genetic distances among the 49 goat breeds. The breeds are highlighted according to their geographic group. See Table 1 for a full definition of the dataset.

Supplementary Table S2 HRR and HRR islands per breed. Each breed reports the number of total HRR by Consecutive Runs (CR) and Sliding Window (SW) methods, the number of islands (HRR islands) and the number of harbored SNPs (SNPs in island). Each statistic is aggregated for geographic group (CODE). See Table 1 for a full definition of the dataset.

**CODE**	**Breed**	**HRR CR**	**HRR SW**	**HRR islands**	**SNPs in island**	**CODE**	**Breed**	**HRR CR**	**HRR SW**	**HRR islands**	**SNPs in island**
Italy	ARG	341	340	2	22	Alpine arch	ALP	520	518	5	50
	ASP	262	261	1	9		BIO	257	255	3	30
	BIA	261	260	5	51		LIV	273	272	3	32
	CAP	294	293	5	53		NVE	219	218	1	10
	FAC	301	300	4	37		ORO	252	252	7	62
	FUL	278	278	4	32		RCC	386	385	2	15
	GAR	389	389	2	22		SAA	544	540	3	30
	GCI	332	332	3	24		VAL	250	250	5	46
	GIR	262	261	2	24		VLS	252	249	5	49
	GRF	326	325	3	26		VPS	273	273	5	51
	JON	178	176	2	20		*total*	*3226*	*3212*	*39*	*375*
	MAL	383	378	3	31						
	MES	275	275	2	21	Africa	ABR	205	204	2	20
	MNT_I	205	205	16	171		BRK	221	220	0	0
	MON	326	326	4	34		GUE	205	204	5	58
	M×S	407	403	4	40		GUM	215	214	3	26
	NIC	250	250	2	26		NBN	187	187	2	21
	RME	344	342	3	34		*total*	*1033*	*1029*	*12*	*125*
	SAR	363	361	4	42						
	TER	339	338	4	40	Asia	BEZ	13	13	1	10
	total	*6116*	*6093*	*75*	*759*		JAT	83	83	2	20
							KAC	53	53	1	10
Europe	CRS	359	358	3	22		KIL	249	249	2	20
	FSS	361	359	2	15		KLS	255	253	2	21
	LNR	345	343	7	69		PAT	126	126	3	26
	MLG	387	385	4	50		TAP	95	95	3	31
	PTV	345	345	4	49		*total*	*874*	*872*	*14*	*138*
	PYR	308	304	1	11						
	total	*2105*	*2094*	*21*	*216*	Brazil	CAN	258	258	5	52

Supplementary Table S3 Gene enrichment analysis based on annotated genes within HRR islands. Per each HRR island, the table reports the type of process (type), the GO and KEGG analysis output (term), genes involved in given term (genes), the significance level (> 0.05) of the gene-term enrichment (p-value), the measure of the enrichment's magnitude (Fold Enrichment) and the correction of significance levels for multiple observations (Bonferroni p-value). For the full definition of breeds, see Table 1. BP = Biological process; CC = Cellular component; MF = Molecular function; KP = KEGG pathway.

HRR island	Type	Term	Genes	*p*-value	Fold enrichment	Bonferroni *p*-value
CHI3-D	BP	GO:0030183~B cell differentiation	*GON4L, YY1AP1*	0.049	35.78	1.000
CHI7-A	BP	GO:0051960~regulation of nervous system development	*LRRTM2, CTNNA1*	0.043	23.38	0.982
CHI7-C	BP	GO:0045935~positive regulation of nucleobase-containing compound metabolic process	*NSD1, UIMC1, FGFR4*	0.038	7.15	0.998
	BP	GO:0051173~positive regulation of nitrogen compound metabolic process	*NSD1, UIMC1, FGFR4*	0.045	6.53	0.999
CHI8-C	BP	GO:0032649~regulation of interferon-gamma production	*CD274, JAK2*	0.012	107.34	0.966
	BP	GO:1903555~regulation of tumor necrosis factor superfamily cytokine production	*CD274, JAK2*	0.017	78.78	0.990
	BP	GO:0046631~alpha-beta T cell activation	*CD274, JAK2*	0.017	78.78	0.990
	BP	GO:0042098~T cell proliferation	*CD274, JAK2*	0.021	64.57	0.996
	BP	GO:0050870~positive regulation of T cell activation	*CD274, JAK2*	0.022	61.34	0.997
	BP	GO:0050670~regulation of lymphocyte proliferation	*CD274, JAK2*	0.023	58.82	0.998
	BP	GO:0032944~regulation of mononuclear cell proliferation	*CD274, JAK2*	0.023	57.25	0.998
	BP	GO:1903039~positive regulation of leukocyte cell-cell adhesion	*CD274, JAK2*	0.024	55.76	0.999
	BP	GO:0070663~regulation of leukocyte proliferation	*CD274, JAK2*	0.025	54.01	0.999
	BP	GO:0022409~positive regulation of cell-cell adhesion	*CD274, JAK2*	0.028	47.44	1.000
	BP	GO:0046651~lymphocyte proliferation	*CD274, JAK2*	0.029	46.42	1.000
	BP	GO:0051251~positive regulation of lymphocyte activation	*CD274, JAK2*	0.029	45.92	1.000
	BP	GO:0045862~positive regulation of proteolysis	*PLGRKT, JAK2*	0.031	42.09	1.000
	BP	GO:0002696~positive regulation of leukocyte activation	*CD274, JAK2*	0.033	40.13	1.000
	BP	GO:0050863~regulation of T cell activation	*CD274, JAK2*	0.034	38.86	1.000
	BP	GO:0050867~positive regulation of cell activation	*CD274, JAK2*	0.035	38.00	1.000
	BP	GO:1903037~regulation of leukocyte cell-cell adhesion	*CD274, JAK2*	0.038	34.91	1.000
	BP	GO:0007159~leukocyte cell-cell adhesion	*CD274, JAK2*	0.042	31.34	1.000
	BP	GO:0051249~regulation of lymphocyte activation	*CD274, JAK2*	0.046	28.91	1.000
	CC	GO:0044459~plasma membrane part	*CD274, PLGRKT, JAK2*	0.014	8.54	0.383
	KP	chx05235:PD-L1 expression and PD-1 checkpoint pathway in cancer	*CD274, JAK2*	0.010	101.10	0.220

CHI11-A	BP	GO:0019219~regulation of nucleobase-containing compound metabolic process	*EFEMP1, PNPT1, PPP4R3B*	0.035	5.35	0.988
CHI11-C	BP	GO:0042472~inner ear morphogenesis	*WDPCP, OTX1*	0.006	159.02	0.377
	BP	GO:0042471~ear morphogenesis	*WDPCP, OTX1*	0.008	130.11	0.439
	BP	GO:0048839~inner ear development	*WDPCP, OTX1*	0.011	88.83	0.572
	BP	GO:0043583~ear development	*WDPCP, OTX1*	0.013	76.22	0.629
	BP	GO:0090596~sensory organ morphogenesis	*WDPCP, OTX1*	0.016	61.05	0.710
	BP	GO:0048562~embryonic organ morphogenesis	*WDPCP, OTX1*	0.018	55.05	0.747
	BP	GO:0048568~embryonic organ development	*WDPCP, OTX1*	0.027	37.66	0.867
	BP	GO:0007423~sensory organ development	*WDPCP, OTX1*	0.032	31.04	0.914
	BP	GO:0048598~embryonic morphogenesis	*WDPCP, OTX1*	0.035	28.43	0.932
CHI12-A	BP	GO:1990349~gap junction-mediated intercellular transport	*GJB2, GJA3, GJB6*	0.000	552.04	0.002
	BP	GO:1902585~single-organism intercellular transport	*GJB2, GJA3, GJB6*	0.000	552.04	0.002
	BP	GO:0051253~negative regulation of RNA metabolic process	*PSPC1, ZMYM5, SAP18, MPHOSPH8*	0.030	5.28	0.999
	BP	GO:0045934~negative regulation of nucleobase-containing compound metabolic process	*PSPC1, ZMYM5, SAP18, MPHOSPH8*	0.043	4.57	1.000
	BP	GO:0031324~negative regulation of cellular metabolic process	*LATS2, PSPC1, ZMYM5, SAP18, MPHOSPH8*	0.047	3.22	1.000
	CC	GO:0005922~connexin complex	*GJB2, GJA3, GJB6*	0.000	120.96	0.019
	CC	GO:0044430~cytoskeletal part	*IFT88, PARP4, LATS2, GJB6, CENPJ, SKA3*	0.020	3.37	0.815
	CC	GO:0098797~plasma membrane protein complex	*GJB2, GJA3, GJB6*	0.023	11.77	0.856
	CC	GO:0044450~microtubule organizing center part	*IFT88, LATS2, CENPJ*	0.025	11.34	0.875
	CC	GO:0015630~microtubule cytoskeleton	*IFT88, PARP4, LATS2, CENPJ, SKA3*	0.031	3.80	0.921
	CC	GO:0005819~spindle	*PARP4, LATS2, SKA3*	0.046	8.09	0.980
	MF	GO:0022829~wide pore channel activity	*GJB2, GJA3, GJB6*	0.000	136.85	0.006
CHI13-B	BP	GO:0051347~positive regulation of transferase activity	*PRNP, PCNA, RASSF2, PRP*	0.003	25.93	0.396

CHI16-D	BP	GO:0061756~leukocyte adhesion to vascular endothelial cell	*SELP*, *SELL*, *SELE*	0.000	234.20	0.003
	BP	GO:0045123~cellular extravasation	*SELP*, *SELL*, *SELE*	0.000	164.44	0.007
	BP	GO:0050900~leukocyte migration	*SELP*, *SELL*, *SELE*	0.002	31.94	0.173
	BP	GO:0007159~leukocyte cell-cell adhesion	*SELP*, *SELL*, *SELE*	0.003	28.21	0.215
	BP	GO:0016339~calcium-dependent cell-cell adhesion via plasma membrane cell adhesion molecules	*SELP*, *SELL*	0.004	429.37	0.290
	BP	GO:0007157~heterophilic cell-cell adhesion via plasma membrane cell adhesion molecules	*SELP*, *SELE*	0.007	234.20	0.467
	BP	GO:0016477~cell migration	*SELP*, *SELL*, *SELE*	0.029	8.18	0.935
	BP	GO:0007596~blood coagulation	*SELP*, *F5*	0.037	42.23	0.970
	CC	GO:0031091~platelet alpha granule	*SELP*, *F5*	0.006	257.04	0.203
	CC	GO:0005887~integral component of plasma membrane	*SELP*, *SELL*, *SELE*	0.024	9.71	0.570
	CC	GO:0031226~intrinsic component of plasma membrane	*SELP*, *SELL*, *SELE*	0.027	9.02	0.622
	MF	GO:0033691~sialic acid binding	*SELP*, *SELE*	0.003	609.94	0.036
	KP	chx04514:Cell adhesion molecules	*SELP*, *SELL*, *SELE*	0.003	26.80	0.058
	KP	chx05144:Malaria	*SELP*, *SELE*	0.029	57.03	0.410
CHI18-A	BP	GO:0033365~protein localization to organelle	*RIPOR1*, *ACD*, *PARD6A*, *NUTF2*, *CTCF*	0.014	4.95	0.998
	BP	GO:0006349~regulation of gene expression by genetic imprinting	*PRMT7*, *CTCF*	0.021	90.08	1.000
	BP	GO:0071514~genetic imprinting	*PRMT7*, *CTCF*	0.027	68.88	1.000
	CC	GO:0005654~nucleoplasm	*EDC4*, *PLA2G15*, *DDX28*, *CENPT*, *ACD*, *PRMT7*, *ESRP2*, *NFATC3*, *NUTF2*, *CTCF*, *THAP11*, *PSKH1*	0.025	1.90	0.863
CHI24-A	BP	GO:0007188~adenylate cyclase-modulating G-protein coupled receptor signaling pathway	*MC2R*, *MC5R*	0.042	39.39	1.000
	BP	GO:0007187~G-protein coupled receptor signaling pathway, coupled to cyclic nucleotide second messenger	*MC2R*, *MC5R*	0.043	38.34	1.000
	MF	GO:0004977~melanocortin receptor activity	*MC2R*, *MC5R*	0.002	889.50	0.015
	MF	GO:0008528~G-protein coupled peptide receptor activity	*MC2R*, *MC5R*	0.029	51.82	0.230
CHI27-B	BP	GO:0018022~peptidyl-lysine methylation	*NSD3*, *ASH2L*	0.041	40.89	0.999
	BP	GO:0016571~histone methylation	*NSD3*, *ASH2L*	0.045	37.17	1.000
	MF	GO:0008170~N-methyltransferase activity	*NSD3*, *ASH2L*	0.045	37.65	0.599
	MF	GO:0008276~protein methyltransferase activity	*NSD3*, *ASH2L*	0.047	35.88	0.617
